# The Nordic Prudent Diet Reduces Risk of Cognitive Decline in the Swedish Older Adults: A Population-Based Cohort Study

**DOI:** 10.3390/nu10020229

**Published:** 2018-02-17

**Authors:** Behnaz Shakersain, Debora Rizzuto, Susanna C. Larsson, Gerd Faxén-Irving, Laura Fratiglioni, Wei-Li Xu

**Affiliations:** 1 Aging Research Center, Department of Neurobiology, Care Sciences and Society, Karolinska Institutet and Stockholm University, 113 30 Stockholm, Sweden; behnaz.shakersain@ki.se (B.S.); Debora.Rizzuto@ki.se (D.R.); laura.fratiglioni@ki.se (L.F.); 2 Unit of Nutritional Epidemiology, Institute of Environmental Medicine, Karolinska Institutet, 171 77 Stockholm, Sweden; susanna.larsson@ki.se; 3 Division of Clinical Geriatrics, Department of Neurobiology, Care Sciences and Society, Karolinska Institutet, 141 57 Huddinge, Sweden; Gerd.Faxen.Irving@ki.se; 4 Stockholm Gerontology Research Center, 113 30 Stockholm, Sweden; 5 Department Epidemiology & Biostatistics, School of Public Health, Tianjin Medical University, Qixiangtai Road 22, Heping District, Tianjin 300070, China

**Keywords:** the Nordic Prudent Dietary Pattern, cognitive function, Population-based cohort study, Nordic countries

## Abstract

Appropriate dietary pattern for preserving cognitive function in northern Europe remains unknown. We aimed to identify a Nordic dietary pattern index associated with slower cognitive decline compared to the Mediterranean-DASH Intervention for Neurodegenerative Delay, Mediterranean Diet, Dietary Approaches to Stop Hypertension, and Baltic Sea Diet indices. A total of 2223 dementia-free adults aged ≥60 were followed for 6 years. Mini-Mental State Examination was administrated at baseline and follow-ups. Dietary intake was assessed by 98-item food frequency questionnaire, and the Nordic Prudent Dietary Pattern (NPDP) was identified. Data were analysed using mixed-effects and parametric survival models and receiver operating characteristic curves with adjustment for potential confounders. Moderate (β = 0.139, 95% CI 0.077−0.201) and high adherence (β = 0.238, 95% CI 0.175−0.300) to NPDP were associated with less cognitive decline compared to other four indices. High adherence to NPDP was also associated with the lowest risk of MMSE decline to ≤24 (HR = 0.176, 95% CI 0.080−0.386) and had the greatest ability to predict such decline (area under the curve = 0.70). Moderate-to-high adherence to the NPDP may predict a better-preserved cognitive function among older adults in Nordic countries. Regional dietary habits should be considered in developing dietary guidelines for the prevention of cognitive impairment and dementia.

## 1. Introduction

Worldwide, the number of people with dementia has reached more than 47 million, with around 37% living in high-income countries [1]. Thus, dementia risk reduction remains a fundamental public health priority. 

Intervention trials have shown associations between the Mediterranean Diet (MedDiet) [2] and the Dietary Approaches to Stop Hypertension (DASH) diet [3] and better cognitive outcomes in Spanish and American populations. Recently, the MIND index (a hybrid Mediterranean-DASH index) was introduced as a dietary pattern that might be associated with reduced neurodegeneration and has been associated with reduced risk of dementia; so far only in one US prospective study [4]. These indices were developed mainly on the basis of general nutrition recommendations or cultural food consumption models. The Nordic multicenter SYSDIET study authors have recently proposed the Baltic Sea Diet (BSD) to quantify healthier dietary choices in the Nordic region [5], which has shown no association with cognition in a Finnish population [6]. 

Socio-cultural values, attitudes, and norms, as well as food palatability, year-round availability, convenience, and affordability may lead to cross-cultural and geographical differences in food consumption [7]. Variations in these factors are postulated as a possible explanation for disparities in scientific findings on diet-cognition associations. For instance, adhering to the traditional Mediterranean diet has been linked to better cognitive outcomes in some North American and south European populations, but not in north Europeans [8]. Therefore, region-specific dietary assessments and modifications for maintaining cognitive performance into advanced ages may be more relevant.

We have previously identified two distinct dietary patterns in Swedish older adults: the prudent and the Western patterns [9]. In the current study, we aimed to: (1) identify a dietary pattern index associated with lower levels of cognitive decline in a Nordic population; (2) compare this association with the association between other dietary indices (MIND, MedDietScore, DASH and BSD) and cognitive decline; and (3) compare how well the five indices predict clinically meaningful decline in global cognitive measures.

## 2. Materials and Methods

### 2.1. Study Design

The Swedish National study on Aging and Care in Kungsholmen (SNAC-K) is an ongoing longitudinal study on a random sample of the community residents aged ≥60 in Kungsholmen, Stockholm, Sweden. Of all the eligible individuals (*n* = 4590), 3363 (73.3%) attended baseline examination (March 2001 to June 2004). The follow-up evaluations of younger participants (aged <78) have been carried out every 6 years, and of older participants (aged ≥78), every 3 years because of greater attrition in older age groups. After excluding people with dementia or missing data on dementia (*n* = 321), people without dementia whose Mini-Mental State Examination (MMSE) scores were missing (*n* = 5) or whose MMSE was <27 (*n* = 306), and those with more than 20% missing data on the semi-quantitative food frequency questionnaire (SFFQ) (*n* = 508), the current study included 2223 adults aged ≥60 who were followed up for 6 years (Supplementary Figure S1).

SNAC-K has been approved by the Regional Ethical Review Board in Stockholm, Sweden, and written informed consent was obtained from all participants at baseline.

### 2.2. Data Collection

Demographic and health-related data were obtained via physician examinations, nurse interviews, and self-administered questionnaires (http://www.snac.org). Educational level was defined as elementary, high school, and university using the reported maximum years of formal schooling as previously described [9]. Civil status was defined as married (included those who were cohabiting), single, widow/divorced. Smoking status was categorized as never-, former- or current-smokers. Physical activity was initially assessed on the basis of WHO and American College of Sports Medicine recommendations and was categorized as (1) inadequate: never, <2–3 times/month, or 2–3 times/month; (2) health-enhancing: light exercise several times/week or every day; and (3) fitness-enhancing: moderate to intense exercise several times/week or every day [10]. 

Chronic disorders were diagnosed by the examining physician on the basis of clinical examination, medical history, laboratory data, and current use of medications. The participants were asked by physicians to show prescription forms and/or the containers for the drugs they used. Drugs were classified on the basis of the Anatomical Therapeutic Chemical (ATC) classification system (e.g., lipid modifying agents: C10; dietary vitamin/minerals supplements: A11 and A12) (http://www.whocc.no/). Information on diseases, diagnosed in accordance with the ninth and tenth revisions of the International Classification of Diseases, was derived from the Swedish Inpatient Registry and included vascular disorders, including heart disease (i.e., coronary heart diseases [ICD-9 codes 410-414; ICD-10 codes I20-I25], atrial fibrillation [ICD-9 code 427.8; ICD-10 code I48], and heart failure [ICD-9 code 428; ICD-10 code I50]), and cerebrovascular disease [ICD-9 codes 430-438; ICD-10 codes I60-I69]; cancer [ICD-9 code 140-239; ICD-10 code C00-D49]; and depression [ICD-9 code 311; ICD-10 code F32]. Hypertension was defined as systolic/diastolic blood pressure of ≥140/90 mmHg or the use of antihypertensive medications (ATC codes C02, C03, and C07) [11]. Diabetes was diagnosed on the basis of medical history, data from the Inpatient Registry [ICD-9 code 250; ICD-10 code E11], use of anti-hyperglycemia medications (ATC code A10), or glycated haemoglobin (HbA1c) >6.4% (46 mmol/mol) [12,13]. In accordance with the National Glycohemoglobin Standardization Program, 1.1% was added to the HbA1c value to equate them to international values [14]. Hypercholesterolemia was defined as non-fasting total plasma cholesterol of ≥6.22 mmol/L (≥240 mg/dL) or use of lipid-lowering medication (ATC code C10) [15]. Body mass index (BMI) was calculated as weight divided by height squared using the nurse-measured weights (kg) and heights (meter). Genomic DNA was also extracted from peripheral blood samples at baseline, and a standard polymerase chain reaction was used for various genotyping, including Apolipoprotein E (APOE) (rs429358) [9]. 

### 2.3. Dementia Diagnosis and Cognitive Function 

A validated clinical three-step procedure [16] was applied to identify prevalent dementia in accordance with the Diagnostic and Statistical Manual of Mental Disorders criteria (4th Edition). Cognitive function was assessed via MMSE for global cognition testing at baseline and each follow-up. Clinically meaningful cognitive decline was defined as a decline to a MMSE score ≤24 [17].

### 2.4. Dietary Assessment

A validated SFFQ with 98 food and beverage items was used at baseline to collect information on habitual dietary intakes [18]. Average frequencies of intakes over the past 12 months for each food item on a 9-level scale (ranging from never to ≥4 times per day) was obtained. Color photos of 4 plates with increasing portions of staple foods (potatoes, rice, and pasta), meat, and vegetables were shown in the SFFQ to estimate portion sizes. For the other food items, standard portion sizes (e.g., the size of an apple as one portion of fruit) were used. Frequencies of food intakes were used in the analyses. The calorie content of each portion was estimated with the National Food Administration’s food composition database using MATs software (Rudans Lättdata, Sweden).

### 2.5. Nordic Prudent Dietary Pattern

In a previous study, we identified two patterns (prudent and Western) associated with the lowest and highest risk of cognitive decline, respectively [9]. In this study, these two dietary patterns were first decomposed into their main food constituents. Then, the independent associations between each food group (and their sub-items) and MMSE change over time were examined. Dietary items that were associated with MMSE change were selected and used in constructing the NPDP. If the direction of the associations was the same for all the sub-items in a food group, the food group was selected. If not, each sub-item was included in the NPDP separately. 

### 2.6. Scoring Dietary Indices 

Four predefined healthy dietary indices including MIND [4], MedDietScore [19], DASH [4], and BSD indices [5], were calculated using the specified food items in the literature. In this study, data were not available on intakes of nuts and olive oil, which was replaced by vegetable oil intake. The consumption of olive oil (monounsaturated fatty acid [MUFA]) is generally low in northern European populations, and vegetable oil intake mainly consists of intake of rapeseed oil, which contains about 30% polyunsaturated fatty acids (PUFAs) and 60% MUFAs [20]. 

The five dietary indices were scored as following: (1) The intakes of food components in each index were dichotomized using the calorie-adjusted and standardized sex-specific population-median of food intakes (frequencies/day) as the cut-off to define low versus high consumption. (2) For the consumption of food items presumed to be healthy, a score of 0 was assigned for intakes below the median, and scores of 1 to 5 were assigned to quintiles of intakes above the median. For the consumption of food components presumed to be less healthy, the scoring was reversed. (3) For BSD, alcohol intake was scored as 1 (>0 to 10 grams/day in women, and >0 to 20 grams/day in men) or 0 (all other amounts) [21]. For other indices, the safe daily intake of wine was defined as >0 to ≤1 drink for women, and >0 to ≤2 drinks for men. (4) The scores assigned to dietary component intake in each index were summed to a total score. Higher score indicated greater adherence to the corresponding diet. 

The index scores were used as continuous and categorical (tertiled as low, moderate, and high adherence) variables in the data analyses. Food/nutrient components of different dietary indices and the sex-specific distribution of index scores are presented in Supplementary Table S1. 

### 2.7. Statistical Analyses

Missing values on the SFFQ and corresponding missing calorie intake values were imputed using the multiple imputation by chained equation. The imputed data were used in the following analyses. Age, sex, education, civil status, smoking, physical activity, BMI, MMSE score, vascular disorders, diabetes, cancer, depression, APOE ε4, dietary supplement use, and total calorie intake were considered major covariates in multiple imputation and in the data analyses.

Inter-correlations between index scores were examined using Pearson’s correlation coefficients. Quantile regression analyses were used to report the difference in median frequency of intakes of different food groups/items by basic characteristics of the study population. The associations of each dietary component and index with MMSE changes were examined using multilevel mixed-effects linear regression. A positive/negative β-coefficient reflected a decrease/an increase in the rate of cognitive decline with more frequent dietary intake or higher index score. In addition to previously mentioned covariates, food items other than the main exposure and components included in each dietary index were entered in the fully adjusted mixed-effects models as separate calorie-adjusted and standardized frequencies of intakes. In supplementary analyses, interactions between time and all additional dietary, and non-dietary covariates were also tested.

In further analyses, hazard ratios (HRs) and 95% confidence intervals (CIs) of MMSE decline to ≤24 in relation to different adherence levels of each dietary index were evaluated using parametric survival models. The cumulative hazard function curves were used to compare the changes in hazard function (MMSE decline to ≤24) over time among the high adherers of different dietary patterns. The ability of each dietary index score to correctly predicting such decline was also assessed by comparing the receiver operating characteristic (ROC) curves based on sensitivity and specificity for every possible cut-off for each dietary index related to cognitive decline. Sensitivity analyses included testing the effect of calorie misreporting and the effect of the imputation procedure on observed associations. All statistical analyses were performed using Stata SE 14 (Stata Corporation, TX, USA). 

## 3. Results

The study population (*n* = 2223) consisted of 871 men (39.2%) and 1352 (60.8%) women, with a mean age of 69.5 ± 8.6 and 71.3 ± 9.1, respectively. Baseline dietary intakes of the study population by age group and sex are presented in Supplementary Table S2. The younger cohorts (<78 years) tended to have higher intakes of non-root vegetables, pasta/rice, vegetable oil, wine, and coffee, and lower intakes of root vegetables, grains/cereals, high-fat dairy products, butter/margarine, and sugar/sweets/pastries than the older cohorts (≥78 years). Women had higher intakes of fruits, vegetables, whole grains, dairy (especially low-fat) products, and lower intakes of red/processed meat, sugar/sweets/pastries, and alcohol than men.

### 3.1. Associations between Individual Food Items and Changes in MMSE 

The annual rates of change in MMSE scores in relation to each specific food group/item are presented in Table 1. On the basis of these results, eight beneficial food items (non-root vegetables, apples/pears/peaches, pasta/rice, poultry, fish, vegetable oil, tea, and water), six less beneficial food items (root vegetables, refined grains/cereals, high-fat dairy products, butter/margarine, sugar/sweets/pastries, and fruit juice), and wine intake were selected to construct the NPDP. 

The highest inter-correlation between index scores was found for MIND and MedDietScore (Pearson’s correlation coefficient = 0.81), whereas the correlation between NPDP and DASH scores was the lowest (Pearson’s correlation coefficient = 0.29). Correlation between NPDP score and MIND, MedDietScore, and BSD was 0.66, 0.64, and 0.47, respectively (Supplementary Table S3). 

### 3.2. Characteristics of the Study Population by Adherence Levels to Different Dietary Patterns

In general, those with high adherence to NPDP, MIND, and MedDietScore were younger, had higher BMI, and a higher proportion were married at baseline. A higher proportion of people with high adherence to all dietary patterns had the highest level of education, were physically very active, and a lower proportion were current-smokers. Those with high adherence to NPDP were less likely to have vascular disorders at baseline (Table 2). 

### 3.3. Association between Dietary Index and Changes in MMSE 

Overall, NPDP scores were more closely associated with cognitive decline than other dietary index scores. In the multi-adjusted mixed-effects models of the categorized index scores, moderate and high adherence to NPDP, and to a lesser extent, moderate and high adherence to MIND were associated with less MMSE decline than low adherence to these dietary patterns. However, only high adherence to MedDietScore was related to less MMSE decline. No associations were observed between either DASH or BSD and MMSE change, except when BSD score was treated as a continuous variable (*p* = 0.049) (Table 3).

### 3.4. Association between Dietary Index and MMSE Decline to ≤24

**Figure 1:**
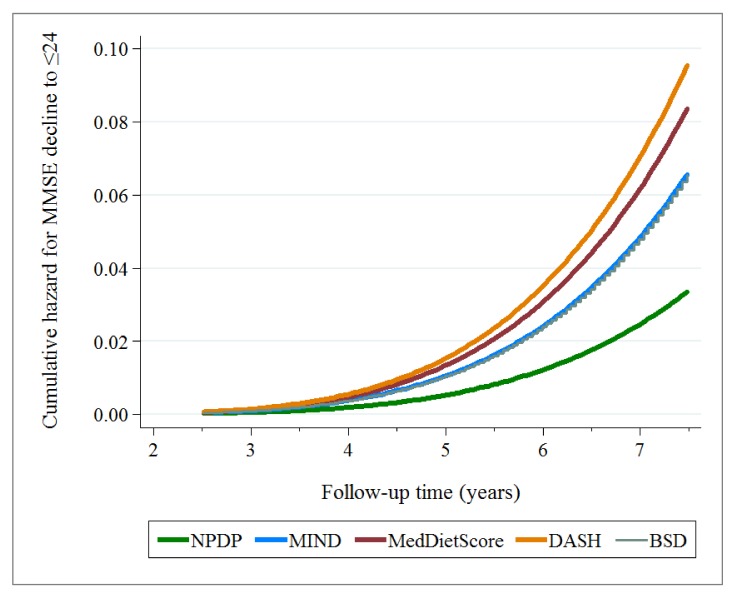
Cumulative hazard function curve (stcurve command in Stata) plotted after running the parametric survival models (Supplementary Table S4) for each dietary index in relation to MMSE decline to ≤24 over 6 years. Each model was adjusted for total calorie intake, age, sex, education, civil status, physical activity, smoking, body mass index, vitamin/mineral supplement intake, vascular disorders, diabetes, cancer, depression, APOE ε4, and dietary components other than those included in each dietary index.

During a mean of 6 years (max 7.5 years, 10,226.3 person-years) of follow-up, 133 incident cases of MMSE decline to ≤24 were identified. In multi-adjusted parametric survival models, those with high adherence to NPDP had the lowest hazard ratio (HR = 0.176, 95% CI 0.080−0.386) for MMSE decline to ≤24 over 6 years (reference: those with a low adherence level). Those with a high adherence to MIND and BSD had an approximately 50% lower risk of MMSE decline to ≤24 that those with low adherence levels. The MedDietScore and DASH showed no associations (Supplementary Table S4). The corresponding cumulative hazard function curves for each dietary index in relation to MMSE decline to ≤24 over 6 years are presented in Figure 1. As it is shown in this plot, the hazard of MMSE decline being experienced by individuals was increasing over time (increasing gradient/slope of the cumulative hazard function). However, the hazard (of MMSE decline) increased the most in those highly adhering to DASH diet, and increased the least in those highly adhering to NPDP.

**Figure 2:**
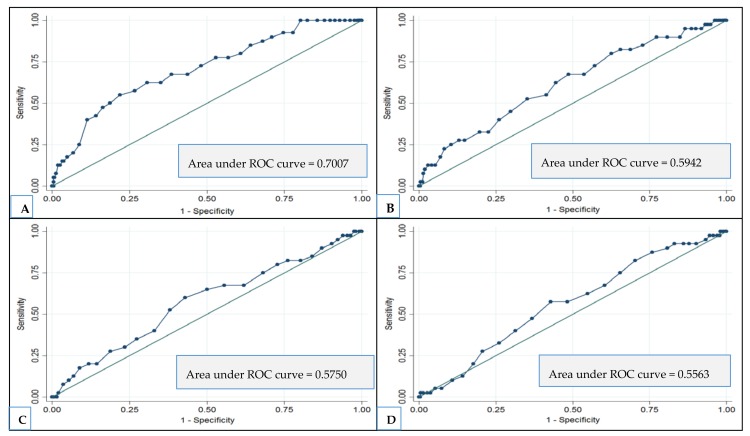
The ability (ROC curve) of each dietary index to correctly predict MMSE decline to ≤24 over 6 years. (**A**) Nordic Prudent Dietary Pattern index; (**B**) MIND index; (**C**) Mediterranean Diet index; (**D**) Baltic Sea Diet index.

In ROC curve analyses for evaluating how well each dietary index score predicts MMSE decline to ≤24, the largest area under the curve was observed for high adherence to NPDP (area under the curve = 0.70) (optimal cutoff point = 31; sensitivity = 70%; specificity = 61%) (Figure 2).

### 3.5. Supplementary Analyses

After adding the interaction terms between time and all non-dietary covariates to the mixed-effects model, high adherence to NPDP remained associated with lower MMSE decline (β = 0.066, 95% CI 0.001−0.130). Adding the interaction terms between time and all dietary covariates other than those included in each dietary index to the mixed-effects models did not change the results (Supplementary Table S5). In a further sensitivity analysis, misreporting of calorie intake was assessed by comparing the reported calorie intakes with age- and sex-specific estimates of energy expenditures using the Oxford equation by Henry [22]. In total, 948 participants (852 who underreported and 96 who over reported) were identified as having misreported calorie intake. A repeated analysis excluding these participants yielded results similar to those of the initial analysis; however, there were no longer any associations between the BSD continuous score and MMSE change. Finally, an analysis restricted to participants with complete data on the SFFQ (*n* = 815) yielded results similar to the original analysis; however, there remained no association between the MedDietScore and MMSE change in the high adherers. All results of the supplementary analyses are available upon request.

## 4. Discussion

In this large population-based cohort study of relatively cognitively intact Swedish older adults, a specific dietary pattern, NPDP, emerged as dietary pattern most closely related to decelerated cognitive decline in this northern European region. Moderate to high adherence to NPDP was more closely associated with less cognitive decline than moderate to high adherence to the other healthful dietary indices, including MIND, MedDietScore, DASH, and BSD. High adherence to NPDP was also associated with the lowest risk of MMSE decline to ≤24, and showed the largest area under the curve representing a better predictor of such decline than the other dietary indices.

The MedDietScore, DASH, and BSD were initially proposed as eating practices with health claims other than prevention of age-related cognitive dysfunction [5,19,23]. Mediterranean diet has been related to a reduced risk of cognitive dysfunction in some North American and Mediterranean populations, but not in Nordic countries [24,25,26]. The MIND index is tailored to promote cognitive function and includes specific vegetables, specific fruit and dairy products, and a “fast/fried foods” component. So far, this hypothesis-driven index has shown associations with less cognitive decline and lower risk of Alzheimer’s disease [4] in North American older adults. 

Despite the advantages of the pre-defined dietary scores, differences in food cultures and resources require tailored dietary pattern assessments to fit local food habits and traditions. For example, olive oil intake is a major component in Mediterranean diet, but in this study (as in other ones) it was replaced by vegetable oil intake, thus causing a deviation from a genuine MedDietScore. Similar concerns apply to other dietary items. Questions arise, therefore, about both the purely scientific and the socioeconomic implications of those pre-defined dietary patterns, such as MeDi, in a northern European or North American country. Because of such disparate trends in food choices and dietary behaviors in different geographical areas, the development and use of local dietary indices for assessing diet-disease associations seem to be logical. In addition, the construction of dietary pattern adherence scores is generally based on relative (within the study population) dietary intakes rather than on absolute intake values, thus inhibiting comparisons across various geographical regions. Moreover, as food intake in older adults could be affected by health conditions in old age (such as physiological anorexia and cachexia), the diet compositions proposed to maintain general health in adults may not be appropriate for maintaining a healthy body and brain at older ages. To the best of our knowledge, thus far no studies have identified dietary patterns that can predict better cognitive function specifically in northern European older populations. In our elderly cohort, we found that people with high adherence to NPDP may have more than 80% reduced risk for MMSE decline to ≤24 than those with the low adherence. Further, the NPDP was the only index with a fair ability (area under the ROC curve = 0.70) to correctly classify the population into those with and without clinically meaningful cognitive decline (MMSE decline to ≤24). 

Previous researchers suggest that to obtain more accurate estimates of the effects of diet on health outcomes, a dietary index should include components with no or low correlation with each other and that are associated with the outcome of interest [27]. In our study, the intra-correlation between NPDP components ranged from −0.25 to 0.28, whereas the intra-correlation between some components in other indices were considerably higher (e.g., 0.85 between E% from saturated fat and E% from total fat in the DASH, or 0.43 between the two vegetable groups in MIND). The authors of earlier studies have also argued that small-scale dietary scores (the 9-item Mediterranean diet scale [28] and BSD score) might not capture extreme levels of intakes leading to overestimation of associations [29]. These methodological differences are one possible explanation for discrepancies between studies using small- and large-scale dietary indices in relation to cognitive health. In this study, the scoring system was purposely applied to create a larger-scale score for each dietary index, although the number of included items slightly differed. 

As it is shown in our study, the effect of high adherence to overall NPDP (β = 0.238; 95% CI = 0.175–0.300) is larger than the effect of almost all its individual food items. What NPDP offers is a list of dietary items which, either in isolation or in combination, have potential cognition protection effect. We believe a “balanced” diet for cognition protection could be defined as combination of any of these suggested foods, although higher variation and adherence to overall NPDP is preferable. Different combination of foods (dietary patterns) may influence cognition through different mechanistic pathways. For example, a high content of omega-3 PUFAs in fish and rapeseed oil have potential neuroprotective properties and can improve the unsaturation index and fluidity in the brain, synaptic and neurotransmitter functioning, and learning and memory performance [30]. In addition, exogenous antioxidants such as dietary vitamin E, vitamin C, carotenoids, and flavonoids are abundant in fruits, vegetables, grains/cereals, legumes, red wine, and tea. Antioxidant-rich diets may reduce the neuronal cell damages caused by free radicals in toxic chain reactions [31]. It is possible that different combination of dietary antioxidant sources in each dietary pattern, such as the unique contribution of apples, pears, wine, and tea in the NPDP, induce distinct effects on brain. In contrast, long-term high consumption of relatively high glycemic index foods (such as refined grains/cereals) may lead to insulin resistance, oxidative stress, and impaired glycemic response, which can negatively affect cognitive function [32]. Emerging evidence has shown that chronic dehydration can influence cognitive performance by affecting different neurotransmitter systems, including serotonin, dopamine, δ-aminobutyric acid, and glutamate levels [33].

The strengths of this study are the large sample size, longitudinal design, high participation rate at follow ups, development of the NPDP, use of a harmonized dietary index scores, and comparison of the locally developed NPDP with “imported” dietary patterns. However, limitations of this study also need to be pointed out. First, the data on habitual dietary intakes were collected using a self-administered SFFQ, which is commonly used in large epidemiological studies. Although the SFFQ used in this study was validated in Swedish populations including older individuals [34], both systematic and random measurement errors might have occurred because the questionnaire may lack some influential food items, and accurate recall is highly dependent on participants’ memory. Previous studies showed that the SFFQ has reasonable reliability and validity in ranking the intake of most foods and nutrients among community-dwelling older adults [35], thus results based on SFFQ data should be interpreted with caution. Second, because of seasonal variation in food availability, changes in personal food preferences or societal eating patterns, and changes in dietary intakes related to health status, it is plausible that the one-time dietary assessment in this study led to attenuated risk estimates. Third, preserved cognitive function was assessed on the basis of repeated measures of MMSE, which is a snapshot of overall cognitive status. Finally, reproducibility and reliability of the NPDP for predicting diet-related cognitive decline in old age needs to be tested in other northern European older populations. 

## 5. Conclusions

The Nordic Prudent Dietary Pattern index is a better predictor of the specific outcome of preserved cognitive function in Swedish older adults than other healthy dietary indices. The NPDP emphasizes high consumption of non-root vegetables, apples/pears/peaches, pasta/rice, poultry, fish, vegetable oils (mainly rapeseed oil), tea, and water and light to moderate wine intake. It discourages high consumption of root vegetables including potatoes, refined grains/cereals, butter/margarine, sugar/sweets/pastries, and fruit juice. This study provides further evidence backing the importance of considering the regional differences when developing healthful dietary scores. The findings from our study could be a basis for future studies aiming to develop dietary guidelines for the prevention of cognitive impairment and dementia. 

## Figures and Tables

**Table 1:** β-coefficients (95% confidence intervals) for the associations between intake of individual dietary items and rate of change in MMSE score over 6 years.

Dietary Items	Model 1—Group Level	Model 2—Subgroup Items
	β (95% CI) ^‡^	β (95% CI) ^‡^
**Vegetables (total)**	0.014 (−0.0002–0.028)	
Non-root vegetables		0.039 (0.021–0.056)
Root vegetables		−0.071 (−0.112–−0.030)
**Fruits (total)**	0.006 (−0.013–0.024)	
Berries		−0.109 (−0.222–0.004)
Apples/pears/peaches		0.051 (0.006–0.096)
Oranges/tangerines/grapefruits		−0.015 (−0.068–0.038)
Bananas		−0.010 (−0.065–0.045)
**Grains/cereals (total)**	−0.021 (−0.035–−0.007)	
Whole grains		−0.015 (−0.036–0.007)
Refined grains/cereals		−0.037 (−0.058–−0.016)
Pasta/rice		0.197 (0.089–0.306)
**Legumes/beans**	0.070 (−0.085–0.225)	
**Red/processed meat**	−0.015 (−0.045–0.015)	
**Poultry**	0.456 (0.226–0.686)	
**Fish**	0.118 (0.013–0.223)	
**Dairy products (total)**	−0.016 (−0.031–−0.001)	
Low-fat dairy products		−0.004 (−0.024–0.016)
Medium-fat dairy products		−0.012 (−0.036–0.013)
High-fat dairy products		−0.056 (−0.093–−0.018)
*Milk*	−0.041 (−0.066–−0.016)	
Low-fat milk		−0.029 (−0.062–0.005)
Medium-fat milk		−0.030 (−0.067–0.005)
High-fat milk		−0.105 (−0.158–−0.051)
*Cheese*	−0.0004 (−0.028–0.028)	
Low-fat cheese		0.020 (−0.019–0.059)
Medium-fat cheese		0.004 (−0.035–0.043)
High-fat cheese		−0.107 (−0.208–−0.006)
*Yoghurt*	0.018 (−0.031–0.067)	
Low-fat yoghurt		−0.010 (−0.075–0.054)
Medium/high-fat yoghurt		0.038 (−0.052–0.127)
*Cream*	0.019 (−0.094–0.132)	0.040 (−0.074–0.154)
**Ice cream**	0.025 (−0.145–0.195)	
**Butter/margarine**	−0.018 (−0.032–−0.004)	
Butter		−0.018 (−0.041–0.004)
Margarine		−0.016 (−0.032–−0.0005)
**Vegetable oil**	0.068 (0.034–0.103)	0.067 (0.033–0.102)
**Sugar/sweets/pastries**	−0.027 (−0.047–−0.008)	
Fast/fried food	−0.042 (−0.169–0.084)	
**Wine**	0.123 (0.054–0.191)	
Red wine		0.102 (0.014–0.190)
White wine		0.172 (0.011–0.333)
**Beer**	0.005 (−0.046–0.055)	
Low-alcohol beer		−0.022 (−0.094–0.049)
Medium-strong beer		0.046 (−0.043–0.135)
Strong beer		0.006 (−0.163–0.175)
**Spirits**	−0.055 (−0.183–0.074)	−0.056 (−0.185–0.073)
**Tea**	0.055 (0.024–0.085)	
**Coffee**	0.017 (−0.007–0.041)	
**Carbonated drinks**	0.028 (−0.077–0.133)	
**Fruit juice**	−0.060 (−0.097–−0.022)	
**Water (plain/mineral)**	0.018 (0.001–0.035)	

Abbreviations: CI, confidence interval; MMSE, Mini-Mental State Examination. The underlined food components are constituents of the Nordic Prudent Dietary Pattern (NPDP) index. A positive β-coefficient reflects a decrease in the rate of cognitive decline with each time increase in daily intake of dietary items. A negative β-coefficient indicates an increase in the rate of cognitive decline with each time increase in daily intake of dietary items.^‡^ Adjusted for total calorie intake, age, sex, education, civil status, physical activity, smoking, body mass index, vitamin/mineral supplement intake, vascular disorders, diabetes, cancer, APOE ε4, and dietary components other than main exposure(s) in each model.

**Table 2:** Baseline characteristics of the study population by lowest (1st tertile) vs. highest (3rd tertile) adherence to different dietary patterns (*n* = 2223).

Characteristics	Dietary Pattern Index Scores (Lowest vs. Highest Tertiles)									
	NPDP		MIND		MedDietScore		DASH		BSD	
	Low (*n* = 720)	High (*n* = 724)	Low (*n* = 799)	High (*n* = 720)	Low (*n* = 774)	High (*n* = 733)	Low (*n* = 812)	High (*n* = 655)	Low (*n* = 805)	High (*n* = 695)
Age (median), years	72.7 (66.3–81.3)	66.2 (60.5–72.3) *	72.2 (62.6–78.4)	66.5 (60.6–76.5) *	72.2 (64.6–78.4)	66.5 (60.7–78.2) *	66.7 (60.7–78.3)	67.5 (60.9–78.2)	66.8 (60.8–78.4)	66.8 (60.8–78.2)
Sex, women	437 (60.6)	442 (61.0)	486 (60.8)	438 (60.8)	475 (61.3)	447 (61.0)	490 (60.3)	399 (61.0)	491 (61.0)	420 (60.4)
Education										
University	193 (26.8)	337 (46.6) *	225 (28.2)	313 (43.5) *	224 (29.0)	304 (41.6) *	279 (34.4)	258 (39.4) *	251 (31.2)	290 (41.7) *
High school	330 (45.8)	300 (41.4)	369 (46.1)	308 (42.7)	360 (46.5)	310 (42.2)	346 (42.5)	287 (43.8)	367 (45.5)	291 (41.9)
Elementary school	197 (27.4)	87 (12.0) *	205 (25.7)	99 (13.8) *	190 (24.5)	119 (16.2) *	187 (23.1)	110 (16.8) *	187 (23.3)	114 (16.4) *
Civil status										
Married	315 (43.7)	417 (57.6) *	374 (46.8)	390 (54.2) *	351 (45.3)	392 (53.4) *	396 (48.8)	351 (53.6)	396 (49.1)	352 (50.7)
Single	124 (17.3)	110 (15.1)	146 (18.3)	104 (14.4)	143 (18.5)	106 (14.5)	139 (17.1)	94 (14.3)	145 (18.0)	110 (15.8)
Widow/divorced	281 (39.0)	197 (27.3) *	279 (34.9)	226 (31.4)	280 (36.2)	235 (32.1)	277 (34.1)	210 (32.1)	264 (32.9)	233 (33.5)
Smoking										
Never	343 (47.7)	291 (40.1) *	330 (41.2)	322 (44.7)	337 (43.5)	326 (44.4)	344 (42.4)	294 (44.9)	335 (41.6)	313 (45.0)
Former	234 (32.5)	347 (47.9) *	307 (38.5)	312 (43.4)	279 (36.0)	335 (45.7)	301 (37.0)	291 (44.4)	306 (38.0)	316 (45.5)
Current	143 (19.8)	87 (12.0) *	162 (20.3)	86 (11.9) *	158 (20.5)	72 (9.9) *	167 (20.6)	70 (10.7) *	164 (20.4)	66 (9.5) *
Physical activity										
Inadequate	194 (26.9)	117 (16.1) *	212 (26.5)	110 (15.3) *	200 (25.9)	113 (15.5) *	220 (27.1)	91 (13.9) *	209 (25.9)	104 (15.0) *
Health-enhancing	401 (55.7)	365 (50.5)	438 (54.8)	360 (50.0)	417 (53.9)	367 (50.0)	431 (53.1)	349 (53.3)	444 (55.2)	352 (50.6)
Fitness-enhancing	125 (17.4)	242 (33.4) *	149 (18.7)	250 (34.7) *	157 (20.2)	253 (34.5) *	161 (19.8)	215 (32.8) *	152 (18.9)	239 (34.4) *
BMI (median), kg/m^2^	25.0 (22.9–27.7)	26.2 (23.8–28.7) *	25.2 (23.0–28.0)	26.0 (23.6–28.5) *	25.2 (23.1–27.8)	25.7 (23.5–28.4) *	25.4 (23.1–28.2)	25.7 (23.5–28.1)	25.3 (23.1–28.0)	25.8 (23.7–28.4)
MMSE (median)	29 (29–30)	29 (29–30)	29 (29–30)	29 (29–30)	29 (29–30)	29 (29–30)	29 (29–30)	29 (29–30)	29 (29–30)	29 (29–30)
Vascular disorders **	646 (89.8)	614 (84.8)*	706 (88.4)	618 (85.8)	676 (87.3)	638 (87.1)	700 (86.2)	572 (87.3)	699 (86.9)	612 (88.0)
Diabetes	240 (33.4)	219 (30.2)	261 (32.6)	228 (31.6)	246 (31.8)	231 (31.5)	269 (33.1)	208 (31.8)	267 (33.2)	218 (31.3)
Cancer	55 (7.7)	47 (6.5)	55 (6.9)	47 (6.5)	53 (6.8)	52 (7.0)	57 (7.0)	50 (7.7)	62 (7.7)	56 (8.0)
Depression	43 (6.0)	32 (4.4)	55 (6.9)	30 (4.2)	47 (6.0)	36 (4.9)	52 (6.4)	33 (5.0)	50 (6.2)	39 (5.6)
Any APOE ɛ4	217 (30.2)	222 (30.6)	234 (29.2)	198 (27.6)	223 (28.9)	218 (29.8)	234 (28.8)	200 (30.5)	225 (28.0)	216 (31.1)
Dietary supplement use	211 (29.3)	197 (27.1)	218 (27.3)	211 (29.2)	214 (27.6)	231 (31.5) *	204 (25.1)	205 (31.3)	206 (25.6)	214 (30.8)

Abbreviations: BMI, body mass index; MMSE, Mini-Mental State Examination; APOE, apolipoprotein E. Values are number (%) for categorical variables, and median (interquartile range) for continuous variables. Number of missing values at baseline: education (1), civil status (3), smoking (13), BMI (26), vascular disorders (1), diabetes (46), depression (6), APOE ε4 (111). Chi-square test for categorical variables, and quantile regression for continuous variables. ** Vascular disorders include hypertension, hypercholesterolemia, cerebrovascular disease (stroke), heart diseases (including coronary heart disease, atrial fibrillation, and heart failure). * *p* < 0.05.

**Table 3:** β-coefficients (95% confidence intervals) for the association between the rate of change in MMSE score over 6 years and the Nordic Prudent Dietary Pattern (NPDP), Mediterranean-DASH Intervention for Neurodegenerative Delay (MIND), Mediterranean Diet Score (MedDietScore), Dietary Approaches to Stop Hypertension (DASH), and Baltic Sea Diet (BSD) indices .

Dietary Index	Continuous Score		Moderate Adherence *		High Adherence *	
	β ^†^ (95% CI)	*p*	β ^†^ (95% CI)	*p*	β ^†^ (95% CI)	*p*
NPDP	0.011 (0.008–0.013)	<0.001	0.139 (0.077–0.201)	<0.001	0.238 (0.175–0.300)	<0.001
MIND	0.006 (0.003–0.009)	<0.001	0.075 (0.012–0.138)	0.019	0.126 (0.064–0.188)	<0.001
MedDietScore	0.006 (0.002–0.009)	0.002	0.063 (−0.002–0.129)	0.057	0.099 (0.036–0.163)	0.002
DASH	0.001 (−0.002–0.004)	0.568	0.015 (−0.056–0.086)	0.673	0.024 (−0.042–0.091)	0.472
BSD	0.004 (0.000–0.008)	0.049	0.018 (−0.060–0.097)	0.645	0.053 (−0.011–0.117)	0.103

Abbreviations: CI, confidence interval; MMSE, Mini-Mental State Examination. * The reference category was those with low adherence. Low, moderate, and high adherence levels to each dietary pattern were respectively defined as the first, second, and third tertile of each total dietary index score. A positive β-coefficient reflects a decrease in the rate of cognitive decline with each unit increase in the dietary index scores (i.e., higher adherence). A negative β-coefficient indicates an increase in the rate of cognitive decline with each unit increase in dietary index scores. ^†^ Adjusted for total calorie intake, age, sex, education, civil status, physical activity, smoking, body mass index, vitamin/mineral supplement intake, vascular disorders, diabetes, cancer, depression, APOE ε4, and dietary components other than those included in each dietary index.

## Supplementary Materials

The following are available online at http://www.mdpi.com/2072-6643/10/2/229/s1, Figure S1: Flowchart of the study population in the Swedish National Study on Aging and Care in Kungsholmen (SNAC-K), Table S1: Dietary components of each dietary index and distribution of total scores for each index in SNAC-K, Table S2: Baseline dietary intake in the SNAC-K population (*n* = 2223), expressed as median frequency of intake of each dietary component (and interquartile range in brackets) per week by age and sex, Table S3: Supplementary Table 3. Inter-correlation between index scores, Table S4: Hazard ratios (95% confidence intervals) for the association between MMSE decline ≤24 over 6 years and the Nordic Prudent Dietary Pattern (NPDP), Mediterranean-DASH Intervention for Neurodegenerative Delay (MIND), Mediterranean Diet Score (MedDietScore), Dietary Approaches to Stop Hypertension (DASH), and Baltic Sea Diet (BSD) indices. Table S5: β-coefficients (95% confidence intervals) for the association between the rate of change in MMSE score over 6 years and the Nordic Prudent Dietary Pattern (NPDP), Mediterranean-DASH Intervention for Neurodegenerative Delay (MIND), Mediterranean Diet Score (MedDietScore), Dietary Approaches to Stop Hypertension (DASH), and Baltic Sea Diet (BSD) indices.
