# Supplementary material for: The Nordic Prudent Diet Reduces Risk of Cognitive Decline in the Swedish Older Adults: A Population-Based Cohort Study
Source: Nutrients. 2018 Feb 17;10(2):229. doi: 10.3390/nu10020229 (PMC5852805; doi:10.3390/nu10020229)
Supplement: Supplementary file 1 [file nutrients-10-00229-s001.docx]

Supplementary Materials:

**Supplementary Table 1.** Dietary components of each dietary index and distribution of total scores for each index in SNAC-K.

| Dietary components | NPDP  15 items | MIND  14 items | MedDietScore  11 items | DASH  10 items | BSD  9 items |
| --- | --- | --- | --- | --- | --- |
| Vegetables | Non-root vegetables | Green leafy vegetables | Potatoes | Total vegetables | Vegetables other than potatoes plus legumes |
|  | Root vegetables | Vegetables other than green leafy | Vegetables other than potatoes |  |  |
| Fruits | Apples/pears/peaches | Berries | Total fruits | Total fruits | Apples/pears/peaches plus berries |
| Grains/cereals | Refined grains/cereals | Whole grains | Whole grains | Total grains/cereals | Oats/rye bread/porridge |
|  | Pasta/rice |  |  |  |  |
| Legumes/beans |  | Legumes/beans | Legumes/beans | Legumes/beans |  |
| Red meat, poultry, fish |  | Red/processed meat | Red/processed meat | Total red meat, poultry, fish | Red/processed meat |
|  | Poultry | Poultry | Poultry |  |  |
|  | Fish | Fish | Fish |  | Fatty fish |
| Dairy products | High-fat dairy products | Cheese | High-fat dairy products | Total dairy products | Milk <2% fat |
| Butter/margarine | Butter/margarine | Butter/margarine |  |  |  |
| Vegetable oil | Vegetable oil | Vegetable oil | Vegetable oil |  |  |
| Sugar/sweets/pastries | Sugar/sweets/pastries | Sugar/sweets/pastries |  | Sugar/sweets/pastries |  |
| Fast/fried food |  | Fast/fried food |  |  |  |
| Alcohol | Wine, drink  F: >0 & ≤1  M: >0 & ≤2 | Wine, drink  F: >0 & ≤1  M: >0 & ≤2 | Wine, drink  F: >0 & ≤1  M: >0 & ≤2 |  | Total alcohol, g  F: >0 & ≤10 g  M: >0 & ≤20g |
| Tea | Tea |  |  |  |  |
| Fruit juice | Fruit juice |  |  |  |  |
|  |  |  |  |  |  |
| Water (plain/mineral) | Water (plain/mineral) |  |  |  |  |
| Other items than food/beverages |  |  |  |  |  |
|  |  |  |  | Sodium |  |
|  |  |  |  | E% from fat | E% from fat |
|  |  |  |  | E% from saturated fat | Fatty acid ratio PUFA:(SFA+Trans)^a^ |
|  |  |  |  |  |  |
| Distribution of index scores |  |  |  |  |  |
| Total possible score | 0 to 71 | 0 to 66 | 0 to 51 | 0 to 50 | 0 to 41 |
| Score range in SNAC-K | 8 to 62 | 4 to 57 | 1 to 44 | 2 to 48 | 0 to 39 |
| Men | 10 to 59 | 4 to 57 | 1 to 44 | 2 to 48 | 1 to 39 |
| Women | 8 to 62 | 7 to 56 | 1 to 42 | 5 to 45 | 0 to 38 |
| Median (IQR) score in SNAC-K | 33 (27, 40) | 30 (24, 36) | 19 (15, 24) | 25 (20, 30) | 16 (12, 21) |
| Men | 33 (27, 41) | 29 (24, 36) | 19 (14, 24) | 25 (20, 30) | 16 (12, 21) |
| Women | 33 (27, 40) | 30 (24, 36) | 19 (15, 24) | 25 (20, 30) | 16 (12, 21) |

Abbreviations: NPDP, Nordic Prudent Dietary Pattern; MIND, Mediterranean-DASH Intervention for Neurodegenerative Delay; MedDietScore, Mediterranean Diet Score; DASH, Dietary Approaches to Stop Hypertension; BSD, Baltic Sea Diet; E%, energy percent; PUFA, polyunsaturated fatty acid; SFA, saturated fatty acid; IQR, interquartile range. ^a^ Ratio between polyunsaturated fatty acids and sum of saturated and trans-fat.

**Supplementary Table 2.** Baseline dietary intake in the SNAC-K population (n=2223), expressed as median frequency of intake of each dietary component (and interquartile range in brackets) per week by age and sex.

| Dietary items | Age | | Sex | |
| --- | --- | --- | --- | --- |
|  | <78 y  *n* = 1516 | ≥78 y  *n* = 707 | Men  *n* = 871 | Women  *n* = 1352 |
| **Vegetables** | 22 (15–30) | 20 (14–29) | 19 (13–27) | 23 ^*^ (16–32) |
| Non-root vegetables | 15 (9–21) | 12 ^*^ (7–20) | 12 (7–19) | 15 ^*^ (9–22) |
| Root vegetables | 7 (4–10) | 8 ^*^ (5–11) | 7 (4–10) | 8 ^*^ (5–10) |
| **Fruits** | 10 (5–17) | 11 (7–17) | 8 (4–15) | 13 ^*^ (7–19) |
| Berries | 1 (1–1) | 1 (1–1) | 1 (0–1) | 1 (1–1) |
| Apples/pears/peaches | 3 (1–7) | 3 (1–7) | 3 (1–7) | 5 ^*^ (2–7) |
| Oranges/tangerines/grapefruits | 3 (1–7) | 3 (1–7) | 1 (1–5) | 3 ^*^ (1–7) |
| Bananas | 3 (1–5) | 3 (1–6) | 1 (1–5) | 3 ^*^ (1–7) |
| **Grains/cereals** | 22 (16–30) | 27 ^*^ (20–37) | 23 (17–34) | 24 (17–32) |
| Whole grains | 10 (7–14) | 14 ^*^ (8–18) | 10 (6–18) | 12 ^*^ (7–17) |
| Refined grains/cereals | 9 (6–14) | 13 ^*^ (8–18) | 10 (6–17) | 9 (6–15) |
| Pasta/rice | 2 (1–3) | 1 ^*^ (1–2) | 2 (1–2) | 2 (1–2) |
| **Legumes/beans** | 1 (1–2) | 1 (1–2) | 1 (1–2) | 1 (1–2) |
| **Red/processed meat** | 8 (5–12) | 8 (5–13) | 9 (6–13) | 7 ^*^ (4–11) |
| **Poultry** | 1 (1–1) | 1 (1–1) | 1 (1–1) | 1 (1–1) |
| **Fish** | 2 (2–3) | 2 (2–3) | 2 (2–3) | 2 (2–3) |
| **Dairy products** | 18 (12–26) | 20 ^*^ (14–29) | 17 (11–26) | 19 ^*^ (13–27) |
| Low-fat dairy products | 6 (1–12) | 6 (1–11) | 4 (1–9) | 7 ^*^ (1–14) |
| Medium-fat dairy products | 7 (2–10) | 7 (3–14) | 7 (3–10) | 7 (3–12) |
| High-fat dairy products | 2 (1–6) | 4 ^*^ (1–8) | 3 (1–7) | 3 (1–7) |
| Milk | 7 (3–9) | 7 (5–14) | 7 (3–10) | 7 (3–12) |
| Low-fat milk | 0 (0–7) | 0 (0–7) | 0 (0–5) | 0 (0–7) |
| Medium-fat milk | 1 (0–5) | 1 (0–7) | 1 (0–5) | 1 (0–7) |
| High-fat milk | 0 (0–1) | 0 (0–3) | 0 (0–1) | 0 (0–1) |
| Cheese | 7 (4–9) | 8 ^*^ (5–13) | 7 (4–10) | 8 ^*^ (5–10) |
| Low-fat cheese | 1 (0–5) | 1 (0–5) | 1 (0–3) | 1 (0–5) |
| Medium-fat cheese | 3 (1–7) | 5 ^*^ (1–7) | 3 (1–7) | 3 (1–7) |
| High-fat cheese | 1 (0–1) | 1 (0–1) | 1 (0–1) | 1 (0–1) |
| Yoghurt | 1 (0–5) | 0 ^*^ (0–5) | 0 (0–4) | 1 ^*^ (0–6) |
| Low-fat yoghurt | 1 (0–3) | 0 ^*^ (0–3) | 0 (0–1) | 1 ^*^ (0–5) |
| Medium/high-fat yoghurt | 1 (0–1) | 0 ^*^ (0–1) | 0 (0–1) | 0 (0–1) |
| Cream | 1 (1–1) | 1 (0–1) | 1 (0–1) | 1 (0–1) |
| **Ice cream** | 1 (0–1) | 1 (0–1) | 1 (0–1) | 1 (0–1) |
| **Butter/margarine** | 14 (7–22) | 20 ^*^ (10–25) | 18 (8–25) | 15 (7–23) |
| Butter | 1 (0–8) | 3 ^*^ (0–14) | 2 (0–10) | 1 (0–8) |
| Margarine | 7 (1–18) | 9 (3–20) | 7 (1–20) | 7 (1–18) |
| **Vegetable oil** | 5 (2–7) | 3 ^*^ (1–6) | 4 (1–7) | 4 (1–7) |
| **Sugar/sweets/pastries** | 7 (3–13) | 10 ^*^ (4–16) | 9 (4–16) | 8 ^*^ (3–13) |
| **Fast/fried food** | 1 (1–2) | 0 ^*^ (0–1) | 1 (1–3) | 0 ^*^ (0–1) |
| **Wine** | 2 (1–4) | 1 ^*^ (0–2) | 2 (1–4) | 1 ^*^ (1–3) |
| Red wine | 1 (1–3) | 0 ^*^ (0–1) | 1 (1–3) | 0 ^*^ (0–3) |
| White wine | 1 (0–1) | 1 (0–1) | 1 (0–1) | 1 (0–1) |
| **Beer** | 1 (0–3) | 0 ^*^ (0–3) | 3 (1–6) | 1 ^*^ (0–2) |
| Low-alcohol beer | 0 (0–1) | 1 ^*^ (0–1) | 1 (0–3) | 0 ^*^ (0–1) |
| Medium-strong beer | 0 (0–1) | 0 (0–1) | 1 (0–1) | 0 ^*^ (0–1) |
| Strong beer | 0 (0–1) | 0 (0–0) | 0 (0–1) | 0 (0–0) |
| **Spirits** | 0 (0–1) | 0 (0–1) | 1 (0–1) | 0 ^*^ (0–1) |
| **Tea** | 3 (1–7) | 3 (0–7) | 3 (0–7) | 3 (1–7) |
| **Coffee** | 18 (7–18) | 7 ^*^ (7–18) | 18 (7–18) | 7 (7–18) |
| **Carbonated drinks** | 0 (0–1) | 0 (0–1) | 0 (0–1) | 0 (0–1) |
| **Fruit juice** | 3 (1–7) | 3 (1–7) | 3 (1–7) | 3 (1–7) |
| **Water (plain/mineral)** | 18 (7–28) | 18 (3–18) | 18 (3–18) | 18 (7–28) |

Abbreviation: SNAC-K, Swedish National study on Aging and Care in Kungsholmen.* *p* < 0.05 derived from quantile regression analyses.

**Supplementary Table 3.** Inter-correlation between index scores.

| Dietary index | NPDP | MIND | MedDietScore | DASH | BSD |
| --- | --- | --- | --- | --- | --- |
| NPDP | 1 |  |  |  |  |
| MIND | 0.66 | 1 |  |  |  |
| MedDietScore | 0.64 | 0.81 | 1 |  |  |
| DASH | 0.29 | 0.54 | 0.59 | 1 |  |
| BSD | 0.47 | 0.67 | 0.74 | 0.75 | 1 |

Numbers are Pearson’s correlation coefficients.

**Supplementary Table 4.** Hazard ratios (95% confidence intervals) for the association between MMSE decline ≤ 24 over 6 years and the Nordic Prudent Dietary Pattern (NPDP), Mediterranean-DASH Intervention for Neurodegenerative Delay (MIND), Mediterranean Diet Score (MedDietScore), Dietary Approaches to Stop Hypertension (DASH), and Baltic Sea Diet (BSD) indices.

| Dietary index | Continuous index score | | Moderate adherence^a^ | | High adherence^a^ | |
| --- | --- | --- | --- | --- | --- | --- |
|  | HR ^b^ (95% CI) | *p* | HR ^b^ (95% CI) | *p* | HR ^b^ (95% CI) | *p* |
| NPDP | 0.950 (0.927 to 0.974) | <0.001 | 0.576 (0.375 to 0.882) | 0.011 | 0.176 (0.080 to 0.386) | <0.001 |
| MIND | 0.965 (0.941 to 0.989) | 0.005 | 0.781 (0.494 to 1.235) | 0.289 | 0.468 (0.261 to 0.840) | 0.011 |
| MedDietScore | 0.982 (0.954 to 1.010) | 0.204 | 1.004 (0.644 to 1.566) | 0.986 | 0.747 (0.448 to 1.245) | 0.263 |
| DASH | 1.000 (0.974 to 1.027) | 0.970 | 1.030 (0.645 to 1.644) | 0.902 | 0.917 (0.543 to 1.548) | 0.746 |
| BSD | 0.961 (0.929 to 0.994) | 0.022 | 0.827 (0.498 to 1.375) | 0.461 | 0.520 (0.284 to 0.952) | 0.034 |

Abbreviations: CI, confidence interval; MMSE, Mini-Mental State Examination. ^a^ The reference category was those with low adherence. Low, moderate, and high adherence levels to each dietary pattern were respectively defined as the first, second, and third tertile of each total dietary index score. ^b^ Adjusted for total calorie intake, age, sex, education, civil status, physical activity, smoking, body mass index, vitamin/mineral supplement intake, vascular disorders, diabetes, cancer, depression, *APOE* ɛ4, and dietary components other than those included in each dietary index.

**Supplementary Table 5.** β-coefficients (95% confidence intervals) for the association between the rate of change in MMSE score over 6 years and the Nordic Prudent Dietary Pattern (NPDP), Mediterranean-DASH Intervention for Neurodegenerative Delay (MIND), Mediterranean Diet Score (MedDietScore), Dietary Approaches to Stop Hypertension (DASH), and Baltic Sea Diet (BSD) indices.

| Dietary index | Moderate adherence * | | High adherence * | |
| --- | --- | --- | --- | --- |
|  | β^†^ (95% CI) | *p* | β^†^ (95% CI) | *p* |
| NPDP | 0.139 (0.077 to 0.201) | <0.001 | 0.239 (0.176 to 0.302) | <0.001 |
| MIND | 0.075 (0.012 to 0.139) | 0.019 | 0.126 (0.064 to 0.188) | <0.001 |
| MedDietScore | 0.062 (−0.005 to 0.127) | 0.070 | 0.098 (0.034 to 0.161) | 0.003 |
| DASH | 0.015 (−0.056 to 0.086) | 0.676 | 0.025 (−0.042 to 0.092) | 0.463 |
| BSD | 0.018 (−0.060 to 0.097) | 0.642 | 0.053 (−0.011 to 0.117) | 0.102 |

Abbreviations: CI, confidence interval; MMSE, Mini-Mental State Examination.* The reference category was those with low adherence. Low, moderate, and high adherence levels to each dietary pattern were respectively defined as the first, second, and third tertile of each total dietary index score. A positive β-coefficient reflects a decrease in the rate of cognitive decline with each unit increase in the dietary index scores (i.e. higher adherence). A negative β-coefficient indicates an increase in the rate of cognitive decline with each unit increase in dietary index scores. ^†^Adjusted for total calorie intake, age, sex, education, civil status, physical activity, smoking, body mass index, vitamin/mineral supplement intake, vascular disorders, diabetes, cancer, depression, *APOE* ε4, dietary components other than those included in each dietary index and their interactions with each other and with time on MMSE.
